# Endoscopic Ultrasound-Guided Gallbladder Drainage for Malignant Distal Biliary Obstruction: Current Evidence, Technical Considerations, and Future Directions

**DOI:** 10.3390/medicina62091726

**Published:** 2026-09-08

**Authors:** Danilo Paduano, Roberto Leone, Gianluca Franchellucci, Francesco Auriemma, Carmine Gentile, Matteo Fiacca, Federica Calabrese, Eleonora Solida, Abed Al-Lehibi, Emad Aljahdali, Abdulrahman Alfadda, Resheed Alkhiari, Ammar Alotaibi, Cesare Hassan, Alessandro Repici, Benedetto Mangiavillano

**Affiliations:** 1 Gastrointestinal Endoscopy Unit, Humanitas Mater Domini, 21053 Castellanza, VA, Italy; francesco.auriemma@materdomini.it (F.A.); carmine.gentile@materdomini.it (C.G.); matteo.fiacca@materdomini.it (M.F.); federica.calabrese@materdomini.it (F.C.); eleonora.solida@materdomini.it (E.S.); benedetto.mangiavillano@mc.humanitas.it (B.M.); 2 IRCCS Humanitas Research Hospital, 20089 Rozzano, MI, Italy; roberto.leone@humanitas.it (R.L.); gianluca.franchellucci@humanitas.it (G.F.); cesare.hassan@hunimed.eu (C.H.); alessandro.repici@hunimed.eu (A.R.); 3 Gastroenterology and Hepatology, King Fahad Medical City, Riyadh 11525, Saudi Arabia; aallehibi@kfmc.med.sa; 4 Department of Medicine, King Abdulaziz University Hospital, King Abdulaziz University, Jeddah 22254, Saudi Arabia; ealjahdli@gmail.com; 5 Section of Gastroenterology, Department of Medicine, King Faisal Specialist Hospital and Research Center, Riyadh 11211, Saudi Arabia; aalfadda@gmail.com; 6 Division of Gastroenterology, Department of Medicine, College of Medicine, King Saud University, Riyadh 11451, Saudi Arabia; ralkhiari@ksu.edu.sa (R.A.); ammalotaibi@ksu.edu.sa (A.A.); 7 Department of Biomedical Sciences, Humanitas University, 20072 Pieve Emanuele, MI, Italy

**Keywords:** endoscopic ultrasound, gallbladder drainage, malignant biliary obstruction, lumen-apposing metal stent, pancreatic cancer, ERCP, choledochoduodenostomy, biliary drainage

## Abstract

Malignant distal biliary obstruction (MDBO) is most commonly managed by endoscopic retrograde cholangiopancreatography (ERCP) with self-expandable metal stent placement. When ERCP fails or is not feasible, endoscopic ultrasound-guided biliary drainage (EUS-BD) has increasingly replaced percutaneous transhepatic biliary drainage in expert centers. Endoscopic ultrasound-guided gallbladder drainage (EUS-GBD) has emerged as an indirect route for biliary decompression when the cystic duct is patent. This comprehensive narrative review focuses on the anatomical rationale, patient selection, procedural technique, comparative positioning, clinical outcomes, adverse events, and unresolved issues of EUS-GBD in MDBO. The supporting evidence is predominantly observational. Published meta-analyses report technical success generally exceeding 90%, pooled clinical success of approximately 82–89%, and overall adverse event rates of approximately 10–14%; these estimates vary with study selection, outcome definitions, assessment time points, and predominantly observational study designs. Comparative studies suggest efficacy and safety similar to EUS-guided choledochoduodenostomy after failed ERCP in anatomically selected patients, although nonrandomized allocation and confounding by indication remain major limitations. A prospective study has demonstrated feasibility as primary palliation, but this strategy cannot yet be considered standard of care. Prophylactic EUS-GBD to prevent post-stenting cholecystitis represents a separate indication and should not be conflated with EUS-GBD for biliary decompression. The key determinant of physiological success is unobstructed communication between the gallbladder and the central biliary tree; therefore, cystic duct patency, tumor relationship to the cystic duct take-off, gallbladder distension, and the absence of extensive gallbladder involvement must be assessed before intervention. EUS-GBD is best positioned as a rescue option after failed ERCP when direct EUS-BD is technically impossible, unsafe, or unsuccessful. Prospective randomized trials, standardized outcome definitions, comparative cost-effectiveness analyses, and dedicated long-term stent management protocols are needed before broader adoption.

## 1. Introduction

Malignant distal biliary obstruction (MDBO) is a frequent complication of pancreatic ductal adenocarcinoma, distal cholangiocarcinoma, ampullary neoplasia, metastatic disease, and less common periampullary malignancies. Biliary decompression is required to treat jaundice and cholangitis, improve pruritus and nutritional status, permit systemic anticancer therapy, and facilitate selected surgical or locoregional treatments. ERCP with transpapillary self-expandable metal stent placement remains the standard first-line approach because it provides internal drainage through a physiological route and is widely available [1,2,3]. Nevertheless, ERCP may fail because of duodenal obstruction, surgically altered anatomy, tumor infiltration of the papilla, the inability to achieve selective biliary cannulation, or failure to traverse a high-grade stricture.

After failed ERCP, traditional alternatives include percutaneous transhepatic biliary drainage (PTBD) and surgical bypass. Both may be effective, but external drainage, catheter-related dysfunction, repeated interventions, impaired quality of life, and procedural morbidity are important limitations. Therapeutic EUS has consequently reshaped the rescue algorithm. Direct EUS-BD can be performed by EUS-guided choledochoduodenostomy (EUS-CDS), hepaticogastrostomy (EUS-HGS), rendezvous, or antegrade techniques. Current European and American guidance supports EUS-BD over PTBD after failed ERCP when local expertise is available [2,3].

EUS-guided gallbladder drainage (EUS-GBD) represents a conceptually different strategy. Rather than accessing the obstructed bile duct directly, it creates a transmural fistula between the gallbladder and the gastric antrum or duodenal bulb. When the cystic duct remains patent and communicates freely with the extrahepatic biliary tree above the malignant obstruction, bile can drain retrogradely through the cystic duct into the gallbladder and then into the gastrointestinal lumen. The first reports were limited to highly selected rescue situations, but subsequent multicenter cohorts, prospective data, comparative studies, and meta-analyses have established EUS-GBD as a clinically relevant option [4,5,6,7,8,9,10,11,12,13,14,15,16,17]. This review critically examines the current evidence and proposes a practical positioning of EUS-GBD within the therapeutic algorithm for MDBO. Prophylactic EUS-GBD performed to prevent cholecystitis after covered transpapillary stenting is conceptually distinct because its target is gallbladder decompression rather than relief of obstructive jaundice; the two indications are therefore considered separately throughout this review.

Beyond summarizing efficacy and safety estimates, the present review is intended to provide an integrated, practice-oriented framework that complements recent systematic reviews and meta-analyses [14,15,16,17,18,19]. Its specific contribution is the combined appraisal of anatomical and physiological prerequisites, the distinction between technical and physiological success, anatomy-based patient selection, comparative positioning of EUS-GBD relative to alternative drainage strategies, a practical clinical algorithm, and a proposed standardized set of biochemical, oncological, and patient-centered outcomes for future studies.

**Figure 1:**
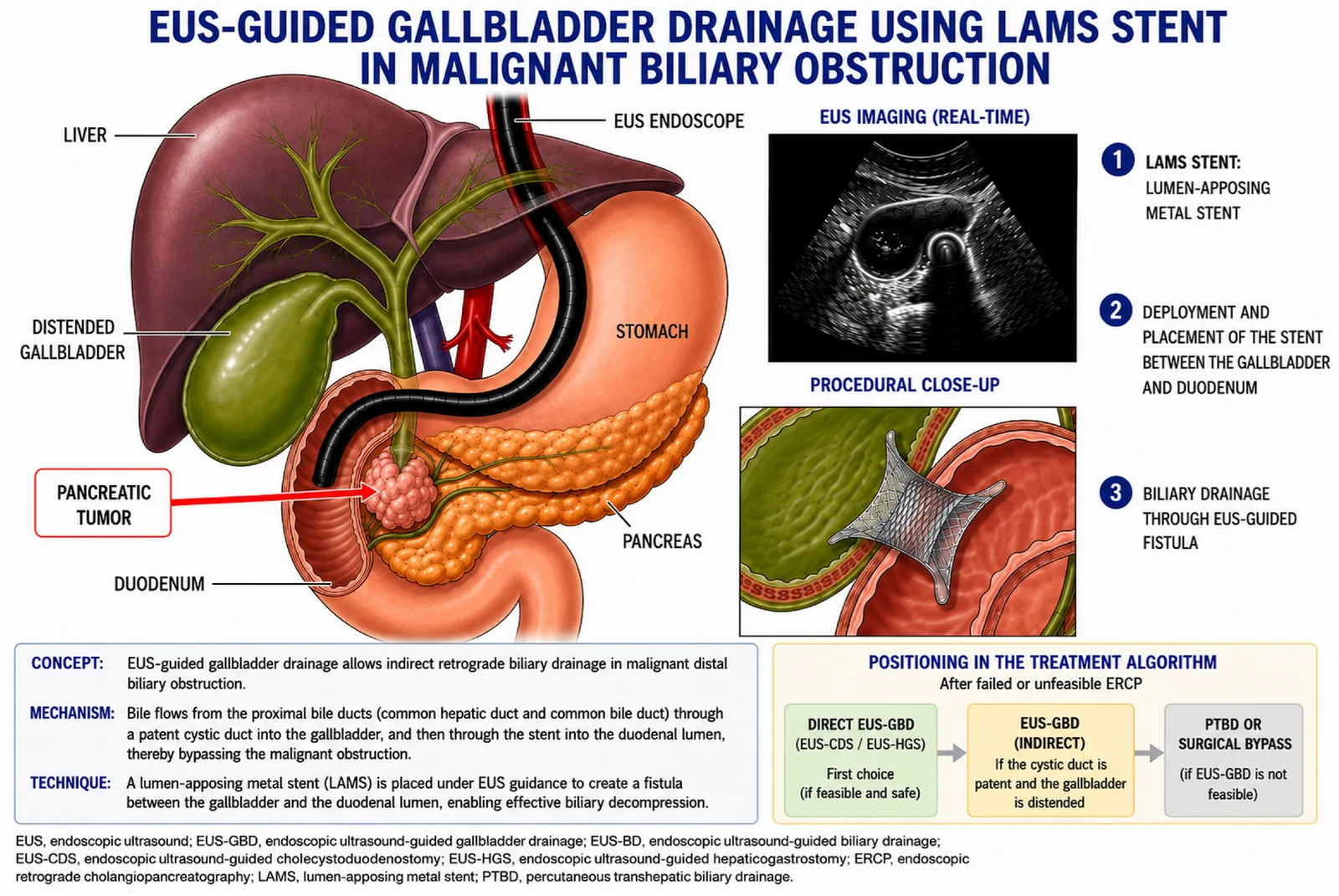
Indirect biliary decompression by EUS-GBD: bile reaches the gallbladder through a patent cystic duct and exits through the transmural stent.

The anatomical mechanism of indirect biliary decompression through a patent cystic duct is illustrated in Figure 1.

## 2. Literature Search and Review Methodology

A comprehensive narrative literature search was performed in PubMed/MEDLINE and supplemented by reference list screening of relevant guidelines, systematic reviews, meta-analyses, and major clinical series. The final PubMed search was updated on 27 August 2026. The principal Boolean strategy was as follows: (“endoscopic ultrasound-guided gallbladder drainage” [Title/Abstract] OR “EUS-GBD” [Title/Abstract]) AND (“malignant biliary obstruction” [Title/Abstract] OR “malignant distal biliary obstruction” [Title/Abstract] OR “distal malignant biliary obstruction” [Title/Abstract] OR “pancreatic cancer” [Title/Abstract] OR “failed ERCP” [Title/Abstract] OR “choledochoduodenostomy” [Title/Abstract]). Additional targeted combinations of these concepts with “lumen-apposing metal stent”, “EUS-guided biliary drainage”, and “clinical success” were used to identify technical and outcome-focused reports. No lower publication-date limit was imposed; publications available up to 27 August 2026 were considered. The search was restricted to English-language records. Full-text studies were prioritized, while clinically relevant PubMed-indexed abstracts were considered when they provided otherwise unavailable data. Conference proceedings and other gray literature were not systematically screened. Literature selection was structured and clinically oriented rather than based on a formal systematic-review protocol. Priority was given to studies specifically addressing EUS-GBD in malignant biliary obstruction, society guidelines, prospective studies, multicenter cohorts, comparative studies, randomized trials, systematic reviews, and meta-analyses. Studies focused exclusively on acute cholecystitis were excluded from the core evidence synthesis unless they informed technical aspects, stent management, or adverse event prevention. No formal PRISMA-based screening process, prespecified risk-of-bias assessment, or quantitative record selection flow was performed. Accordingly, this article was designed as a comprehensive narrative review and should not be interpreted as a systematic review or meta-analysis; the possibility of selective citation inherent to narrative synthesis is acknowledged as a limitation.

## 3. Anatomical and Physiological Rationale

### 3.1. The Gallbladder as an Indirect Route for Biliary Decompression

The effectiveness of EUS-GBD for MDBO depends on the gallbladder functioning as a low-pressure reservoir that remains connected to the common hepatic duct through a patent cystic duct. Following creation of a cholecystoenteric fistula, bile produced by the liver must flow from the intrahepatic ducts to the common hepatic duct, through the cystic duct, into the gallbladder, and across the transmural stent. This pathway is longer and more anatomically variable than direct EUS-CDS. It is therefore vulnerable to tumor infiltration, cystic duct obstruction, inflammatory stenosis, gallstones, viscous bile, or unfavorable cystic duct angulation.

Historical surgical and ERCP-based observations demonstrated that cystic duct patency is maintained in a substantial proportion of patients with malignant obstructive jaundice, but patency cannot be assumed solely from gallbladder distension. A distended gallbladder may support the feasibility of puncture, yet it may coexist with partial or complete cystic duct obstruction. Conversely, a nondistended gallbladder can be technically difficult to access and may indicate inadequate inflow. Cross-sectional imaging, diagnostic EUS, previous cholangiography, and, when available, contrast-enhanced assessment should be integrated before selecting EUS-GBD [20,21,22].

### 3.2. Determinants of Cystic Duct Patency

Tumor level and extension are central. Lesions confined below the cystic duct take-off are theoretically favorable, whereas hilar extension, direct involvement of the cystic duct orifice, gallbladder neck invasion, or extensive hepatoduodenal ligament infiltration reduce the probability of effective decompression. Covered transpapillary metal stents may themselves obstruct the cystic duct orifice, particularly when the tumor already involves this region. Prior cholecystectomy obviously precludes EUS-GBD. Additional unfavorable factors include severe gallbladder wall thickening, extensive ascites, intervening varices or vessels, gallbladder malignancy, and a gallbladder completely filled with stones or tumor.

## 4. Indications and Patient Selection

### 4.1. Rescue Drainage After Failed ERCP

The strongest current indication is rescue palliation of MDBO after unsuccessful or impossible ERCP, particularly when EUS-CDS or EUS-HGS is not feasible, has failed, or is judged to carry excessive risk. Common scenarios include a nondilated common bile duct, an inaccessible papilla because of malignant duodenal obstruction, an unstable endoscope position, intervening vessels, surgically altered anatomy, or failed direct bile duct puncture. EUS-GBD may be completed during the same anesthetic session, avoiding PTBD and an external catheter [4,5,6,7,9,10].

### 4.2. EUS-GBD as an Alternative to Direct EUS-BD

EUS-CDS is generally favored when the extrahepatic bile duct is dilated and accessible because it directly drains the target system. However, the common bile duct may be too small for safe LAMS deployment, and tumor or vascular anatomy may make the puncture trajectory unsuitable. In such patients, a distended gallbladder closely apposed to the duodenal bulb or gastric antrum can offer a technically easier target. Comparative observational data suggest broadly similar efficacy between EUS-GBD and EUS-CDS in selected patients, but these findings should be interpreted in light of nonrandomized allocation and anatomical selection [11,13,18].

### 4.3. Primary Biliary Drainage

A prospective multicenter study evaluated EUS-GBD as the initial approach for jaundice palliation in unresectable MDBO and reported technical success of 100% and clinical success of 100% [8]. These data demonstrate proof of concept but do not establish equivalence or superiority to ERCP. Primary EUS-GBD should therefore remain investigational or be limited to exceptional circumstances in expert centers, such as an inaccessible papilla with a clearly patent cystic duct and a highly favorable gallbladder position. The irreversible creation of a cholecystoenteric fistula, uncertain implications for subsequent surgery, and the availability of established transpapillary or direct EUS-BD techniques argue against routine first-line use [23,24].

### 4.4. Prophylactic Gallbladder Drainage

A separate indication is prevention of post-stenting cholecystitis in patients with malignant involvement of the cystic duct orifice undergoing covered metal stent placement. In a randomized trial, prophylactic EUS-GBD reduced acute cholecystitis in this high-risk population [25]. This strategy should not be conflated with EUS-GBD for jaundice: the therapeutic target is gallbladder decompression rather than drainage of the intrahepatic biliary system. Moreover, prophylactic EUS-GBD introduces procedural risk in patients who may never develop cholecystitis, and editorials have appropriately urged caution [26,27]. Patient selection should therefore be stringent and individualized.

## 5. Preprocedural Assessment

Preprocedural planning should combine clinical, oncological, radiological, and endoscopic information. Contrast-enhanced computed tomography or magnetic resonance imaging should define the level of biliary obstruction, tumor relationship to the cystic duct insertion, gallbladder size, gallstones, ascites, collateral vessels, and potential gastric or duodenal windows. The intended oncological pathway must be clarified, including resectability, expected survival, chemotherapy plans, and the possibility of later surgical bypass or pancreatic resection.

Diagnostic EUS immediately before drainage is essential. The operator should confirm a sufficiently distended gallbladder, identify the neck and body, choose the shortest stable avascular trajectory, and assess whether the duodenal bulb or gastric antrum provides the better window. Doppler examination is mandatory. When cystic duct patency is uncertain, prior cholangiography, gallbladder opacification, contrast-enhanced EUS, or intraprocedural contrast injection after puncture may provide additional information, although none is perfectly reliable. Antibiotic prophylaxis is generally appropriate because the procedure accesses an obstructed and potentially colonized biliary compartment.

Practical preprocedural checklist for probable cystic duct patency and EUS-GBD candidacy: (1) the malignant obstruction is distal to the cystic duct take-off and there is no direct tumor involvement of the cystic duct or gallbladder neck on CT/MRI; (2) the gallbladder is present, sufficiently distended, and not extensively replaced by tumor or packed with stones; (3) cross-sectional imaging shows no obvious cystic duct interruption and, when available, prior ERCP/cholangiography demonstrates gallbladder opacification or preserved communication; (4) diagnostic EUS confirms a short, stable, avascular gastric or duodenal window and excludes major neck/hilar infiltration, intervening vessels, and clinically significant ascites; and (5) when patency remains uncertain, low-pressure intraprocedural contrast assessment may be considered, but forceful injection solely to prove communication should be avoided. This checklist is pragmatic rather than validated and should be integrated with the overall oncological context and local expertise.

## 6. Procedural Technique

### 6.1. Access Route

EUS-GBD can be performed through the duodenum (cholecystoduodenostomy) or stomach (cholecystogastrostomy). The transduodenal route is often preferred because the duodenal bulb is relatively fixed and usually lies close to the gallbladder neck, potentially reducing stent movement and food reflux. The transgastric route may provide a more stable endoscope position or a better window to the gallbladder body in some patients. Route selection should be based on the shortest distance, absence of intervening vessels, scope stability, and the ability to deploy both stent flanges without tension.

### 6.2. Lumen-Apposing Metal Stents

Electrocautery-enhanced lumen-apposing metal stents (EC-LAMSs) have simplified EUS-GBD by enabling single-device puncture and deployment. The distal flange is released under EUS guidance within the gallbladder, followed by controlled apposition and deployment of the proximal flange in the gastrointestinal lumen. Freehand deployment is widely used by experts, while a guidewire-assisted approach may be selected in difficult anatomy. Commonly used diameters range from 8 to 15 mm. In expert practice, smaller diameters may reduce the size of the fistulous tract and theoretically limit food reflux, whereas larger diameters provide more rapid drainage, facilitate endoscopic re-access, and may be less susceptible to occlusion by debris; the trade-off is greater tissue apposition and a larger permanent fistulous tract. No randomized evidence defines the optimal diameter for MDBO [28,29]. Data from acute cholecystitis suggest that multiple LAMS sizes can be used effectively, but these observations should be extrapolated cautiously because malignant biliary decompression depends on sustained cystic duct inflow rather than treatment of an infected gallbladder alone [28,29].

Coaxial placement of a double-pigtail plastic stent through the LAMS is practiced variably. Potential advantages include reduced food impaction, less direct contact between the distal flange and gallbladder wall, and maintenance of a drainage channel if the LAMS lumen becomes partially obstructed. Potential disadvantages are added cost, procedural complexity, and an additional device that may itself migrate or occlude. Experience in acute cholecystitis and consensus practice supports selective rather than universal use [28,29], but evidence specific to malignant biliary decompression is insufficient to recommend routine coaxial plastic stenting.

**Figure 2:**
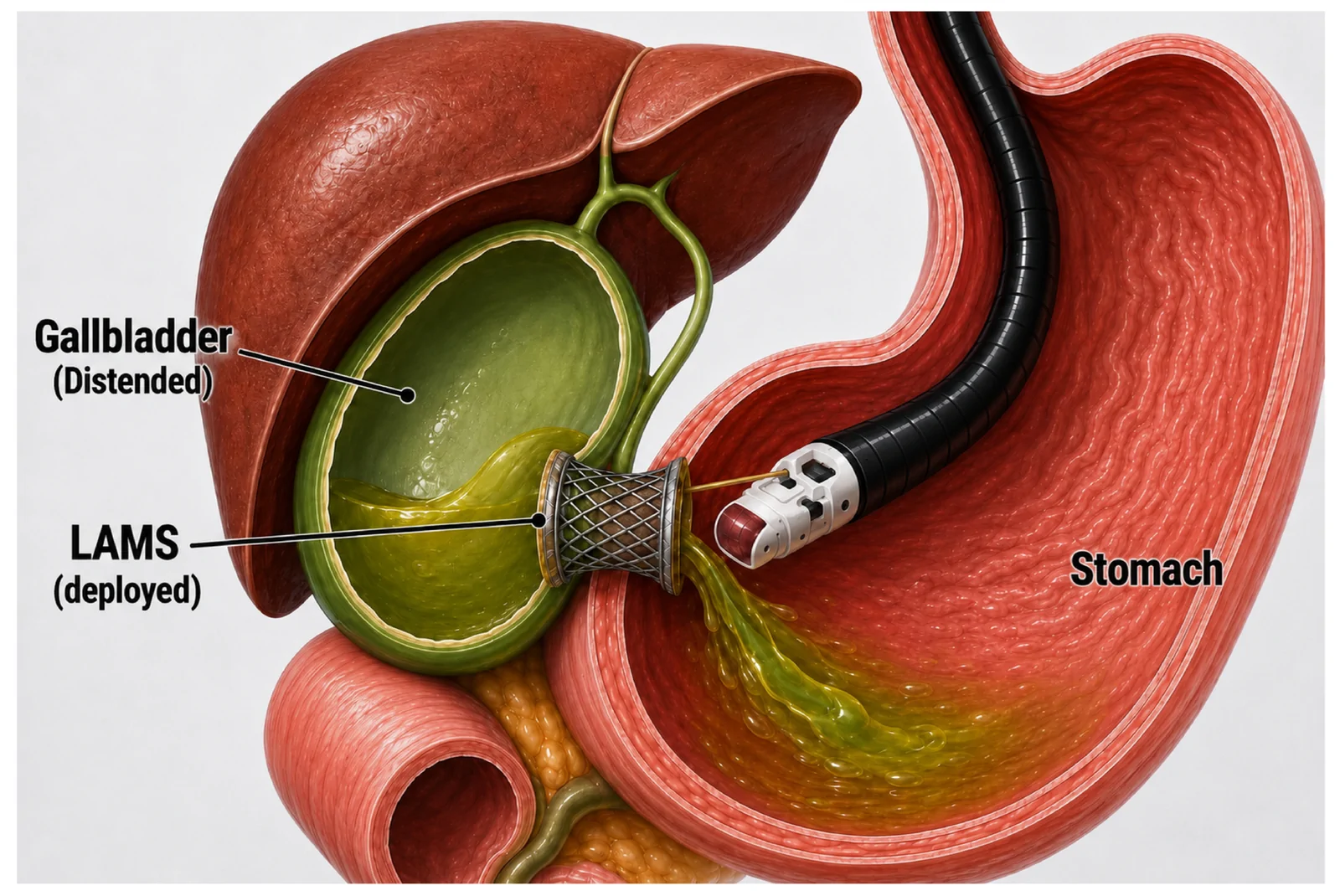
A schematic representation of lumen-apposing metal stent deployment during EUS-guided cholecystogastrostomy. The distal flange is deployed within the gallbladder and the proximal flange within the gastric lumen, creating a stable cholecystogastric fistula and allowing for internal drainage of bile.

The final configuration of the LAMS after EUS-guided cholecystogastrostomy is illustrated in Figure 2.

### 6.3. Confirmation of Effective Biliary Communication

After stent deployment, bile flow through the LAMS is reassuring but does not prove that the entire biliary tree will decompress. When safe, low-pressure contrast injection through the stent can document filling of the cystic duct and common hepatic duct; forceful or high-pressure injection should be avoided. If contrast findings are inconclusive or injection is considered unsafe, confirmation should rely on integration of preprocedural CT/MR imaging, EUS assessment of the cystic duct region, prior cholangiographic information when available, and the early biochemical trajectory. Clinical monitoring must include bilirubin, cholestatic enzymes, fever, abdominal pain, and evidence of cholangitis. Failure of bilirubin to decline should prompt early reassessment for cystic duct obstruction, stent dysfunction, undrained intrahepatic segments, or progressive hepatic failure.

**Figure 3:**
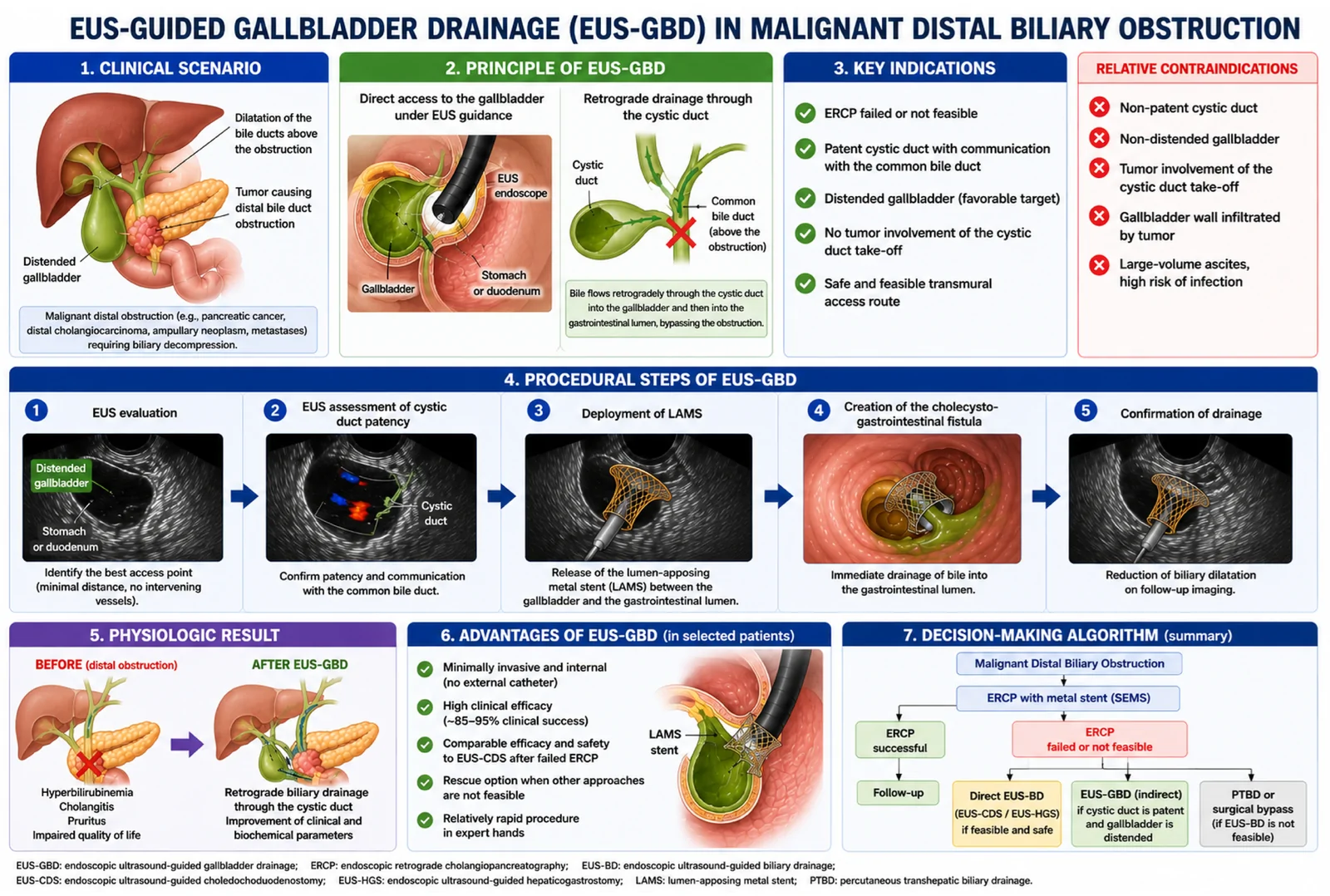
Practical overview of anatomical eligibility, patient selection, and main procedural steps of EUS-GBD after failed or unfeasible ERCP.

The principal patient selection criteria and procedural sequence for EUS-guided gallbladder drainage are summarized in Figure 3.

## 7. Clinical Evidence

### 7.1. Early Feasibility and Rescue Series

The initial report by Itoi and colleagues described EUS-guided cholecystogastrostomy as an alternative extrahepatic drainage route in pancreatic cancer with duodenal invasion [12]. Imai et al. subsequently evaluated EUS-GBD as rescue therapy after unsuccessful ERCP and demonstrated that the approach could relieve malignant obstructive jaundice in carefully selected patients [4]. Chang et al. reported a case series using a LAMS, further supporting procedural feasibility [5]. These early studies established the central concept that the gallbladder can serve as a “backdoor” to the biliary tree when conventional access routes fail [20].

Larger multicenter experiences followed. Issa et al. reported successful rescue EUS-GBD in unresectable malignant biliary obstruction across multiple centers [6]. Binda et al. published a large multicenter LAMS series with 100% technical success and high clinical success, supporting reproducibility in experienced units [7]. More recent registry and cohort data from Europe, North America, and Australasia have confirmed high technical and clinical success, relatively short hospitalization, and the possibility of resuming oncological treatment after drainage [9,10,30,31,32].

### 7.2. Prospective Evidence

The prospective study by Mangiavillano et al. is notable because EUS-GBD was used as the first approach rather than as a salvage procedure. Technical success was achieved in all patients, and clinical success was reported in 100% [8]. Although encouraging, the study involved highly selected patients treated by expert endoscopists and lacked a randomized comparator. It therefore supports feasibility and hypothesis generation rather than routine replacement of ERCP.

### 7.3. Comparative Studies

The GALLBLADEUS study compared EUS-CDS and EUS-GBD after failed ERCP and found that both strategies were effective in selected patients [13]. A subsequent international comparative study similarly reported comparable efficacy and safety, while emphasizing that EUS-GBD requires a gallbladder in situ and clear cystic duct patency [11]. These studies suggest that EUS-GBD may be a legitimate alternative when direct bile duct drainage is unfavorable, but only in carefully selected patients with favorable gallbladder anatomy and a patent cystic duct. Confounding by indication is substantial: patients selected for EUS-GBD generally have a larger, more accessible gallbladder and preserved cystic duct communication, whereas those selected for EUS-CDS require a sufficiently dilated common bile duct and an acceptable duodenal puncture window. Importantly, the comparative studies did not systematically report the denominator of patients anatomically eligible for both procedures; therefore, the degree of true cross-eligibility cannot be quantified from the published data. Propensity matching can reduce measured baseline imbalance but cannot fully correct this anatomy-driven treatment assignment. Randomized or pragmatic prospective comparisons with prespecified anatomical eligibility are needed.

### 7.4. Systematic Reviews and Meta-Analyses

Meta-analyses published in 2023–2025 consistently show high technical and clinical success. Kamal et al. reported pooled clinical success of 85% and adverse events of 13% for rescue EUS-GBD [15]. Osman et al. reported pooled technical success of 92.1%, clinical success of 81.6%, and adverse events of 13.8% [14]. Rizzo et al. reported pooled clinical success of 89% overall (88% in full-text studies), reintervention of 8%, and overall adverse events of 10% [16]. Khoury et al. reported pooled technical success of 99.2%, clinical success of 88.1%, and adverse events of 13.7% [17]. These estimates are reassuring but remain limited by overlapping cohorts, small sample sizes, predominantly retrospective designs, heterogeneous definitions of clinical success, and variable follow-up [19].

Taken together, the strength of evidence differs substantially across clinical scenarios. Rescue EUS-GBD after failed or impossible ERCP is supported by multiple multicenter observational cohorts, prospective data, and meta-analyses and therefore represents the best-established indication. Use as an alternative to EUS-CDS is supported mainly by comparative observational studies and meta-analytic evidence in anatomically selected patients. Primary EUS-GBD is supported by limited prospective evidence and remains investigational or highly selective. Prophylactic EUS-GBD is supported by randomized evidence in a specific high-risk population with cystic duct orifice involvement, but its therapeutic target is prevention of cholecystitis rather than decompression of the intrahepatic biliary tree.

## 8. Summary of Key Clinical Studies

The principal clinical studies evaluating EUS-GBD in malignant distal biliary obstruction and their key numerical outcomes are summarized in Table 1.

**Table 1:** Summary of key clinical studies evaluating EUS-guided gallbladder drainage in malignant biliary obstruction, including sample size, stent type, and principal numerical outcomes.

Study/Design (N)	Stent	Technical/Clinical Success	Adverse Events	Reintervention/Key Limitation
Imai et al. [4]; single-center rescue cohort (*n* = 12)	Transmural stent (pre-EC-LAMS)	100%/91.7%	16.7%	Single-center, small cohort; pre-EC-LAMS era
Chang et al. [5]; rescue case series (*n* = 9)	EC-LAMS	100%/77.8%	0%	Small case series; no comparator; 1/9 required radiologic drainage
Issa et al. [6]; multicenter rescue cohort (*n* = 28)	LAMS 93%; SEMS 7%	100%/93%	17.9% delayed AEs	Retrospective selected rescue cohort after failed ERCP/EUS-BD; no comparator
Binda et al. [7]; 14-center rescue cohort (*n* = 48)	LAMS	100%/81.3%	10.4%	Retrospective expert-center cohort; no randomized comparator
Mangiavillano et al. [8]; prospective primary-drainage study (*n* = 37)	EC-LAMS	100%/100%	10.8%	Prospective single-arm study; highly selected patients; no control group
Debourdeau et al. [13]; multicenter EUS-GBD vs. EUS-CDS (EUS-GBD *n* = 41; total *n* = 78)	LAMS	100%/87.8%	9.8% peri-procedural; 7.3% late	Nonrandomized comparison; confounding by indication; cross-eligibility not reported
Martínez-Moreno et al. [10]; 9-center rescue registry (*n* = 96)	Hot Axios LAMS	99%/78.1%	26.3%	Retrospective multicenter registry; no comparator; 57.1% of eligible patients started chemotherapy
Chieng et al. [9]; two-center salvage cohort (*n* = 26)	LAMS (8–15 mm)	100%/100%	50% delayed; 15.4% serious	Small two-center cohort; high delayedAE burden; 15.4% reintervention
Mangiavillano et al. [11]; 28-center EUS-GBD vs. EUS-CDS (EUS-GBD *n* = 136; PSM *n* = 112)	LAMS	97.3%/83.0%	19.6%	Observational propensity-matched comparison; residual anatomical selection; cross-eligibility not reported
Goudot et al. [33]; GALLBLADEUS-2 international 17-center retrospective cohort (*n* = 166)	EC-LAMS	98.8%/82.9%	25.9% any AE; 23.5% high-grade morbidity (AGREE ≥ IIIa)	Retrospective multicenter cohort; no comparator; 12-month patency 70.4%; ascites predicted dysfunction
Robles-Medranda et al. [25]; randomized prophylaxis trial (EUS-GBD *n* = 22; total *n* = 44)	LAMS after ERCP SEMS	NR/not a jaundice drainage endpoint	Acute cholecystitis 0% vs. 22.7% control	Different therapeutic target (prevention of cholecystitis rather than jaundice relief)

Abbreviations: EC-LAMS, electrocautery-enhanced lumen-apposing metal stent; ERCP, endoscopic retrograde cholangiopancreatography; EUS-BD, endoscopic ultrasound-guided biliary drainage; EUS-CDS, endoscopic ultrasound-guided choledochoduodenostomy; EUS-GBD, endoscopic ultrasound-guided gallbladder drainage; LAMS, lumen-apposing metal stent; AE, adverse event; PSM, propensity score matching; SEMS, self-expandable metal stent; NR, not reported. Note: In the comparative EUS-GBD versus EUS-CDS studies, the proportion of patients anatomically eligible for both techniques was not systematically reported; this limits quantification of cross-eligibility and residual selection bias.

## 9. Clinical Success: Definitions and Interpretation

Clinical success has been defined variably as a reduction in serum bilirubin of at least 50%, normalization or near-normalization of bilirubin, relief of cholangitis, or a composite outcome assessed at time points ranging from 3 days to several weeks. This heterogeneity complicates cross-study comparison. A rapid 50% decline within 3 days may be unrealistic in patients with severe cholestasis, hepatic metastases, sepsis, or impaired liver reserve, whereas a late endpoint can misclassify early procedural failure. Future studies should report absolute and relative bilirubin change at standardized intervals, resolution of cholangitis, ability to initiate or resume anticancer therapy, length of stay, reintervention, stent patency, quality of life, and survival [9,34].

For future prospective studies, a minimum standardized outcome set should include (1) technical success; (2) confirmation or strong evidence of effective biliary communication through the cystic duct; (3) absolute and relative bilirubin change at prespecified time points, including day 7, day 14, and day 30; (4) a ≥50% reduction in bilirubin by day 7–14 when clinically appropriate, together with durability of response at day 30; (5) resolution of cholangitis; (6) recurrent biliary obstruction and reintervention at 30 and 90 days; (7) ability and time to initiate or resume systemic anticancer therapy; (8) adverse events graded according to a standardized classification; and (9) stent patency, hospitalization, patient-reported quality of life, and survival. Reporting this core set would improve comparability between studies and distinguish purely technical deployment from clinically meaningful biliary decompression.

Clinical failure despite technically successful deployment may result from an unrecognized cystic duct obstruction, tumor progression, insufficient drainage of the intrahepatic system, food or sludge occlusion, LAMS malposition, or nonobstructive causes of persistent jaundice. We therefore propose that technical success require correct transmural LAMS deployment with immediate access to the gallbladder, whereas physiological success should additionally require demonstrated or highly credible communication with the central biliary tree, a clinically meaningful bilirubin decline at standardized early time points, resolution of cholangitis when present, and no need for unplanned rescue biliary drainage. Durable physiological success should also be documented at 30 days when survival permits.

## 10. Adverse Events and Their Management

### 10.1. Intraprocedural Events

Potential intraprocedural adverse events include bleeding, perforation, pneumoperitoneum, bile leak, stent misdeployment, and injury to adjacent organs. Catastrophic misdeployment can occur if the distal flange is released outside the gallbladder or if the gallbladder separates from the gastrointestinal wall during deployment. Rescue options include immediate bridging with a fully covered metal stent, placement of a second LAMS, endoscopic closure, radiological drainage, or surgery. These procedures should therefore be performed in centers with interventional radiology and surgical backup.

### 10.2. Early and Late Events

Early events include abdominal pain, fever, cholecystitis, cholangitis, bleeding, bile peritonitis, and stent occlusion. Late events include food impaction, buried stent, migration, recurrent biliary obstruction, gallbladder mucosal injury, and tumor-related loss of cystic duct patency. Pooled adverse event rates are approximately 10%, although definitions and grading vary [16,17]. Reintervention is usually endoscopic and may involve LAMS clearance, coaxial plastic stent placement or exchange, placement of an additional metal stent, or conversion to EUS-CDS, EUS-HGS, or PTBD.

### 10.3. Stent Removal or Indefinite Indwell?

The optimal duration of LAMS indwell in malignant biliary drainage is unknown. In patients with limited life expectancy and durable function, indefinite indwell may avoid an additional procedure. In patients with longer survival, prolonged contact may increase the risk of buried stent, tissue overgrowth, food reflux, or delayed bleeding. Evidence beyond 6–12 months is particularly sparse, and no validated surveillance or elective-removal strategy exists for patients with prolonged survival. Removal could eliminate the drainage route and precipitate recurrent jaundice if cystic duct-dependent decompression remains necessary. A pragmatic approach is symptom- and liver-test-based surveillance integrated with oncological follow-up, with a low threshold for cross-sectional imaging and endoscopic reassessment when biochemical deterioration, cholangitis, pain, or suspected stent dysfunction occurs. Planned reassessment may be reasonable in selected patients expected to survive long enough for late adverse events to become clinically relevant. Prospective studies should explicitly report stent management and long-term patency.

## 11. EUS-GBD Versus Alternative Drainage Strategies

The main advantages, limitations, and patient-burden considerations of the available drainage strategies for malignant distal biliary obstruction are summarized in Table 2.

**Table 2:** Comparison of available drainage strategies for malignant distal biliary obstruction, including patient burden and quality of life considerations.

Strategy	Advantages/Current Position	Main Limitations	Patient Burden/QoL Considerations
ERCP	Physiological transpapillary route; broad availability; first-line standard when papilla and stricture are accessible	Cannulation failure; pancreatitis; inaccessible papilla	Internal drainage; usually low device-related burden when successful
EUS-CDS	Direct extrahepatic internal drainage; preferred rescue EUS-BD when a dilated distal CBD is safely accessible	Requires sufficiently dilated CBD; bile leak or misdeployment risk	Avoids external catheter care; direct comparative QoL data remain limited
EUS-HGS	Useful with duodenal obstruction or altered anatomy; alternative rescue route using left intrahepatic ducts	Technically complex; bile leak, peritonitis, stent migration	Internal drainage but potentially greater procedural burden and need for expert follow-up
EUS-GBD	Large accessible target; useful when CBD is small; rescue alternative in a distended gallbladder with patent cystic duct	Indirect route; requires gallbladder in situ, cystic duct patency, and favorable access	Avoids external catheter; burden depends on LAMS patency, cystic duct integrity, and reintervention need
PTBD	Widely available and effective when endoscopic approaches are unavailable or unsuitable	External catheter; discomfort; dislodgement; repeat procedures	High day-to-day device burden from catheter care, dislodgement risk, and exchanges; comparative QoL data remain limited
Surgical bypass	Potentially durable drainage in selected fit patients or during concomitant surgery	High morbidity and delayed recovery; rarely used for isolated palliation	Higher initial treatment burden and recovery time; may provide durable drainage in selected patients

Abbreviations: CBD, common bile duct; ERCP, endoscopic retrograde cholangiopancreatography; EUS-BD, endoscopic ultrasound-guided biliary drainage; EUS-CDS, endoscopic ultrasound-guided choledochoduodenostomy; EUS-GBD, endoscopic ultrasound-guided gallbladder drainage; EUS-HGS, endoscopic ultrasound-guided hepaticogastrostomy; PTBD, percutaneous transhepatic biliary drainage.

In expert centers, the practical hierarchy after failed ERCP is determined by anatomy rather than by a rigid sequence. Direct drainage should generally be preferred when a safe target is available. EUS-GBD becomes particularly attractive when the common bile duct is not sufficiently dilated, EUS-CDS deployment is unsafe, left intrahepatic ducts are unsuitable for EUS-HGS, or previous direct EUS-BD has failed. PTBD remains essential when endoscopic options are unavailable or contraindicated.

## 12. Proposed Clinical Algorithm

A practical, anatomy-driven approach to the selection of biliary drainage strategies in malignant distal biliary obstruction is summarized in Algorithm 1. The proposed pathway integrates ERCP feasibility, anatomical suitability for EUS-guided drainage, cystic duct patency, gallbladder accessibility, and the availability of local expertise.


**Algorithm 1.** Proposed anatomy-driven clinical decision-making algorithm for biliary drainage in malignant distal biliary obstruction.1. Confirm unresectable or palliative MDBO and need for biliary decompression. 2. Perform ERCP with metal stent placement when the papilla is accessible and cannulation is feasible. 3. After failed or impossible ERCP, assess EUS anatomy in the same session when expertise is available. 4. Prefer direct EUS-BD (usually EUS-CDS) when the bile duct is sufficiently dilated and a safe avascular window exists. 5. Before rescue EUS-GBD, obtain multidisciplinary input when performance status is borderline, expected survival is uncertain, surgery or other oncological interventions remain possible, or more than one rescue route is technically feasible. 6. Consider EUS-GBD when: (a) the gallbladder is present and adequately distended; (b) cystic duct patency is highly likely or demonstrated; (c) the tumor does not occlude the cystic duct insertion; (d) a short stable transduodenal or transgastric window exists; and (e) direct EUS-BD is impossible, unsafe, or unsuccessful. 7. Use EUS-HGS or PTBD when EUS-GBD criteria are not met. 8. Monitor bilirubin and clinical response early; reassess promptly if cholangitis persists or bilirubin fails to decline. 9. Reserve primary EUS-GBD and prophylactic EUS-GBD for carefully selected patients in expert centers or clinical studies.


**Figure 4:**
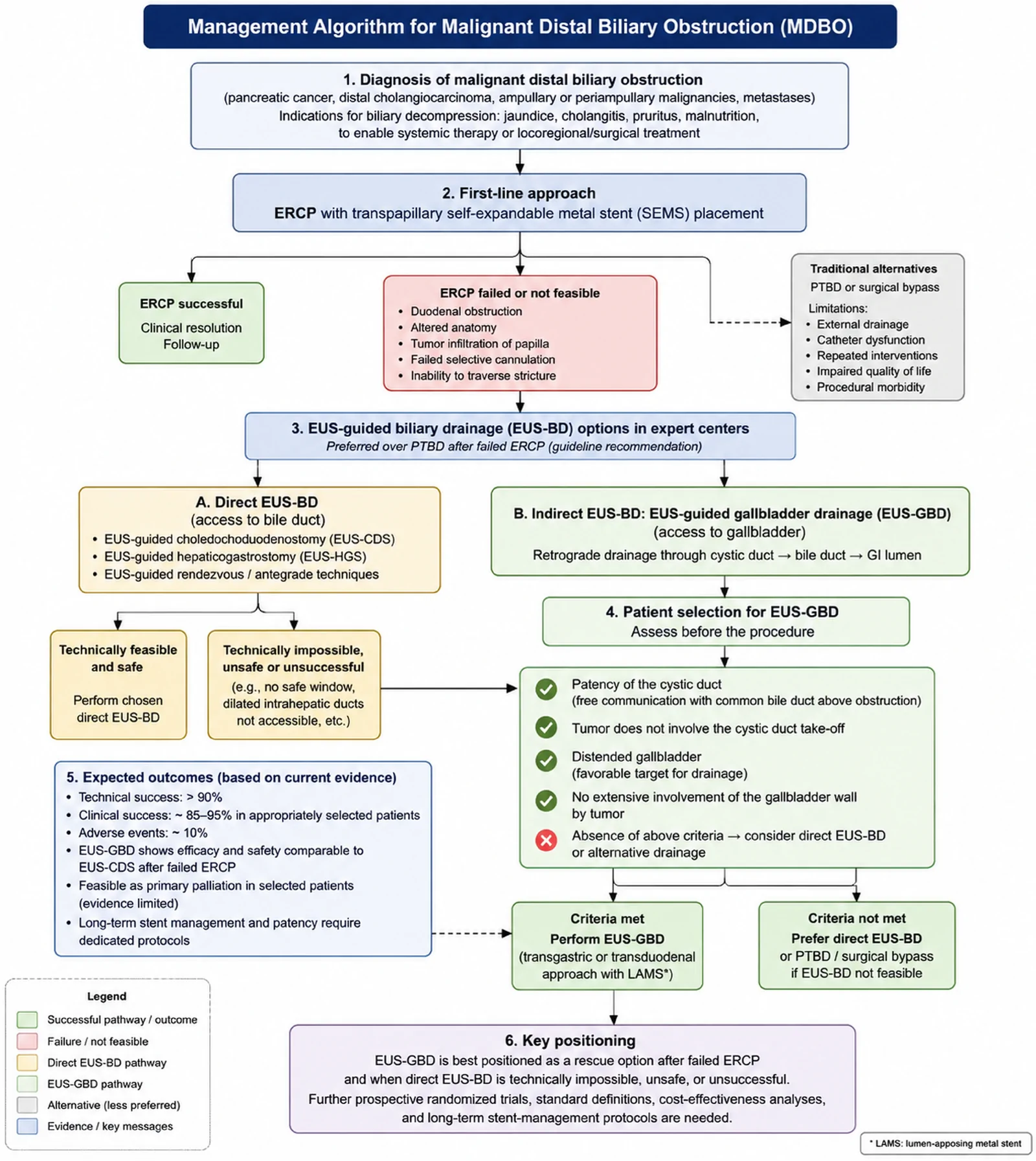
The clinical decision-making algorithm for malignant distal biliary obstruction. ERCP remains the first-line approach. After failed or unfeasible ERCP, direct EUS-guided biliary drainage should generally be preferred when technically feasible and safe. EUS-guided gallbladder drainage may be considered as a rescue option in selected patients with a distended gallbladder, a patent cystic duct, no tumor involvement of the cystic duct take-off, and a favorable transmural access route. ERCP, endoscopic retrograde cholangiopancreatography; EUS-BD, endoscopic ultrasound-guided biliary drainage; EUS-GBD, endoscopic ultrasound-guided gallbladder drainage; PTBD, percutaneous transhepatic biliary drainage; LAMS, lumen-apposing metal stent.

The proposed decision-making pathway for the management of malignant distal biliary obstruction, including the positioning of EUS-GBD after failed ERCP, is summarized in Figure 4.

## 13. Training, Expertise, and Implementation

EUS-GBD should be performed by therapeutic endoscopists experienced in EUS-BD, LAMS deployment, management of misdeployment, and endoscopic treatment of adverse events. Competence derived from EUS-GBD for acute cholecystitis is helpful but does not replace understanding of malignant biliary anatomy and cystic duct physiology [35,36]. Institutional pathways should include anesthesia support, interventional radiology, hepatopancreatobiliary surgery, and access to urgent cross-sectional imaging. Centers without immediate interventional radiology or surgical rescue capability should not undertake complex rescue EUS-GBD unless a predefined rapid transfer pathway is in place. We propose a minimum procedural data set documenting the following: indication and prior drainage attempts; tumor level and resectability; preprocedural evidence for cystic duct patency; gallbladder size and access route; Doppler findings and target wall distance; LAMS type and diameter; freehand versus guidewire-assisted deployment; the use of a coaxial plastic stent; the method used to assess biliary communication; immediate bile flow; technical and physiological success; adverse events with standardized grading; bilirubin at day 7, day 14, and day 30 when available; 30- and 90-day reintervention; and the ability to initiate or resume anticancer therapy [29,34].

Centralization is important because the relevant patients are often frail, septic, and undergoing time-sensitive oncological care. A failed attempt can delay chemotherapy and expose the patient to multiple procedures. Multidisciplinary discussion should therefore focus not only on technical feasibility but also on expected survival, performance status, goals of care, and the probability that bilirubin reduction will translate into meaningful oncological benefit.

## 14. Evidence Gaps and Future Directions

The principal evidence gap is the absence of adequately powered randomized trials comparing EUS-GBD with EUS-CDS, EUS-HGS, and PTBD after failed ERCP. Trials should stratify by bile duct diameter, duodenal obstruction, tumor type, cystic duct anatomy, and prior metal stenting. Such trials are difficult because only a minority of patients at any single center are anatomically eligible for more than one rescue technique, and urgent biliary decompression often limits time for trial enrollment. Multicenter pragmatic designs, central eligibility definitions, and prespecified anatomy-based strata may therefore be more feasible than unrestricted randomization.

A standardized method for assessing cystic duct patency is urgently needed. Future studies should compare computed tomography, magnetic resonance cholangiopancreatography, ERCP findings, contrast-enhanced EUS, and intraprocedural cholecystography against subsequent clinical success. Predictive models could combine tumor location, gallbladder volume, cystic duct diameter, bilirubin level, and imaging characteristics.

Other priorities include consensus definitions of technical and clinical success; patient-reported outcomes; time to initiation or resumption of chemotherapy; the proportion of patients able to receive planned oncological therapy; recurrent hospitalization; 30- and 90-day reintervention; stent patency; quality of life; treatment-related burden; cost-effectiveness; optimal LAMS diameter; the role of coaxial plastic stents; antibiotic strategy; surveillance; and management of patients with unexpectedly long survival. These oncological and patient-centered endpoints are particularly important because successful biliary drainage is clinically meaningful only when it translates into symptom control, avoidance of repeated procedures or hospitalization, and timely access to anticancer treatment. The role of EUS-GBD as primary drainage requires direct comparison with ERCP and primary EUS-CDS. Prophylactic EUS-GBD also warrants further study to identify patients whose cholecystitis risk is sufficiently high to justify an additional invasive intervention. Existing Italian consensus guidance already positions EUS-GBD as a rescue option in selected MDBO when other EUS-BD approaches are not feasible [37]. A more focused consensus process dedicated to EUS-GBD in MDBO may nevertheless be warranted to harmonize cystic duct assessment, technical terminology, physiological success criteria, long-term stent management, and oncological outcome reporting.

## 15. Discussion

EUS-GBD has evolved from an improvised rescue maneuver into an evidence-supported component of the therapeutic armamentarium for MDBO. Its major strength is the ability to exploit a large and often easily visualized target when the bile duct cannot be safely accessed. EC-LAMS technology permits rapid single-session internal drainage and can avoid the burden of external percutaneous catheters. Across meta-analyses, pooled clinical success is approximately 82–89% and overall adverse event rates are approximately 10–14%; comparative observational studies suggest outcomes broadly similar to EUS-CDS in anatomically selected patients [11,13,14,15,16,17,18,33].

Its fundamental limitation is that it remains an indirect drainage route: technically successful LAMS deployment does not guarantee decompression if the cystic duct is functionally obstructed. This distinguishes EUS-GBD from EUS-CDS and makes anatomical selection central to outcome. A large or easily punctured gallbladder alone is insufficient; a credible physiological pathway from the intrahepatic ducts to the gallbladder must be established.

Comparative studies indicating equivalence with EUS-CDS are clinically important but should not be overinterpreted. Their results apply to anatomically selected patients, and allocation bias may favor each technique in its optimal setting. Rather than identifying a universally superior procedure, current evidence supports an anatomy-tailored strategy. EUS-CDS remains the most direct option for a dilated extrahepatic duct, EUS-HGS is valuable when the duodenum is inaccessible or intrahepatic access is preferable, and EUS-GBD provides a complementary route when the gallbladder–cystic duct axis is favorable.

The first-line prospective data are provocative. Avoiding papillary manipulation could theoretically reduce pancreatitis and simplify drainage in patients with duodenal invasion. However, primary EUS-GBD creates a nonphysiological fistula, depends on an anatomically variable cystic duct, and may complicate future surgery. Until randomized evidence demonstrates a clear patient-centered advantage, its use should remain selective. Similarly, prophylactic EUS-GBD may prevent cholecystitis in a high-risk subgroup but exposes all treated patients to EUS-GBD-related risk. These two emerging applications should be considered distinct research domains rather than extensions of established rescue practice [8,25,26,27].

The updated literature search identified the 2026 GALLBLADEUS-2 international multicenter study, which provides additional information on predictors of EUS-GBD dysfunction. In 166 patients, technical and clinical success were 98.8% and 82.9%, respectively, with 12-month biliary patency of 70.4%. Ascites was the only independent predictor of drainage dysfunction, whereas EUS-confirmed cystic duct patency was independently associated with clinical success. Neither access route nor LAMS diameter significantly influenced biliary patency, although 15 mm LAMSs were associated with fewer high-grade adverse events. These findings reinforce the importance of patient-level anatomy and physiology while highlighting the need for prospective validation [33].

This review is limited by the quality of the underlying evidence. Most studies are retrospective, sample sizes are modest, outcome definitions differ, and some cohorts may overlap. In addition, the structured literature search was limited to PubMed/MEDLINE; although supplemented by reference-list screening of relevant guidelines, reviews, meta-analyses, and major clinical series, studies indexed exclusively in other bibliographic databases may have been missed. Because study selection and synthesis were narrative rather than protocol-driven, selective citation cannot be fully excluded. Procedures were generally performed at high-volume centers by experts, limiting generalizability. Publication bias favoring technically successful or positive early series is also plausible. Nevertheless, the concordance of technical, clinical, and safety outcomes across geographically diverse cohorts supports the validity of EUS-GBD as a rescue option when its anatomical prerequisites are respected.

## 16. Conclusions

EUS-GBD is an effective and relatively safe method of internal biliary decompression in selected patients with MDBO. Its best-established role is rescue therapy after failed or impossible ERCP when direct EUS-BD is not feasible, unsafe, or unsuccessful. A favorable transmural window and, most importantly, a patent cystic duct not compromised by tumor are essential prerequisites. Comparative observational data suggest outcomes similar to EUS-CDS in appropriately selected anatomy but do not justify replacing direct bile duct drainage when that approach is readily feasible. Primary and prophylactic applications remain selective, and future prospective studies should use standardized anatomical, biochemical, oncological, and patient-centered outcome definitions.

## Data Availability

No new data were created or analyzed in this narrative review. Data sharing is not applicable to this article.
